# COVID-19 vaccine hesitancy in underserved communities of North Carolina

**DOI:** 10.1371/journal.pone.0248542

**Published:** 2021-11-01

**Authors:** Irene A. Doherty, William Pilkington, Laurin Brown, Victoria Billings, Undi Hoffler, Lisa Paulin, K. Sean Kimbro, Brittany Baker, Tianduo Zhang, Tracie Locklear, Seronda Robinson, Deepak Kumar

**Affiliations:** 1 Julius L. Chambers Biomedical Biotechnology Research Institute, North Carolina Central University, Durham, NC, United States of America; 2 Division of Research and Sponsored Programs, North Carolina Central University, Durham, NC, United States of America; 3 Department of Mass Communication, North Carolina Central University, Durham, NC, United States of America; 4 Department of Nursing, North Carolina Central University, Durham, NC, United States of America; 5 Department of Pharmaceutical Sciences, North Carolina Central University, Durham, NC, United States of America; 6 Department of Public Health Education, North Carolina Central University, Durham, NC, United States of America; University of Arkansas for Medical Sciences, UNITED STATES

## Abstract

**Background:**

In the United States, underserved communities including Blacks and Latinx are disproportionately affected by COVID-19. This study sought to estimate the prevalence of COVID-19 vaccine hesitancy, describe attitudes related to vaccination, and identify correlates among historically marginalized populations across 9 counties in North Carolina.

**Methods:**

We conducted a cross-sectional survey distributed at free COVID-19 testing events in underserved rural and urban communities from August 27 –December 15, 2020. Vaccine hesitancy was defined as the response of “no” or “don’t know/not sure” to whether the participant would get the COVID-19 vaccine as soon as it became available.

**Results:**

The sample comprised 948 participants including 27.7% Whites, 59.6% Blacks, 12.7% Latinx, and 63% female. 32% earned <$20K annually, 60% owned a computer and ~80% had internet access at home. The prevalence of vaccine hesitancy was 68.9% including 62.7%, 74%, and 59.5% among Whites, Blacks, and Latinx, respectively. Between September and December, the largest decline in vaccine hesitancy occurred among Whites (27.5 percentage points), followed by Latinx (17.6) and only 12.0 points among Blacks. 51.2% of respondents reported vaccine safety concerns, 23.7% wanted others to get vaccinated first, and 63.1% would trust health care providers about the COVID-19 vaccine. Factors associated with hesitancy in multivariable logistic regression included being female (OR = 1.90 95%CI [1.36, 2.64]), being Black (OR = 1.68 1.16, 2.45]), calendar month (OR = 0.76 [0.63, 0.92]), safety concerns (OR = 4.28 [3.06, 5.97]), and government distrust (OR = 3.57 [2.26, 5.63]).

**Conclusions:**

This study engaged the community to directly reach underserved minority populations at highest risk of COVID-19 that permitted assessment of vaccine hesitancy (which was much higher than national estimates), driven in part by distrust, and safety concerns.

## Introduction

The COVID-19 pandemic in the United States has exacerbated deeply rooted socioeconomic and health disparities in historically marginalized populations [1, 2]. Since the coronavirus epidemic started in the US, it has become one of the leading cause of death [3, 4]. COVID-19 incidence is disproportionately higher among both Blacks and Hispanics. Racial minorities are more likely to become severely ill, and nearly three times as likely as Whites to die from COVID-19 [5].

In November 2020, Moderna and Pfizer-BioNTech released their findings from randomized trials of COVID-19 vaccines, showing a remarkable 90–95% efficacy [6, 7]. The Food and Drug Administration (FDA) issued Emergency Use Authorizations (EUA) for the Pfizer-BioNTech vaccine on December 10, 2020 [8] and the Moderna vaccine on December 18, 2020 [9]. The Advisory Committee on Immunization Practices (ACIP) updated recommendations for allocating initial supplies with tiered distribution to groups at highest risk first (e.g., health care workers, nursing home residents, and the elderly) [10, 11]. Since that time, the vaccine has been distributed throughout the United States. The population effectiveness [12] to reach herd immunity thresholds for COVID-19 requires an estimated 70% of the population to be vaccinated [13, 14]. To promote vaccine uptake and access requires resources, strategies and structural intervention in multiple sectors [15]. Vaccine hesitancy is defined as a “delay in acceptance or refusal of vaccination despite availability of vaccination services [16].” The decision to accept, delay, or refuse vaccination is complex depending heavily on the context, place, and the specific vaccine. It is paramount that we understand COVID-19 vaccine hesitancy in historically marginalized populations (HMPs) where underlying trust issues directly impact vaccine decisions.

Since early in the pandemic, several studies have used commercial panels, address lists, or random telephone surveys, representative of the US population to estimate vaccine hesitancy [17–29]. The advantage of panel studies is that a large sample can be enrolled within a very short time (weeks or days), but participation often requires internet access. Two studies conducted during May and June, 2020 drawing from different panels, each estimated that 31% of the US population had vaccine hesitancy [23, 27]. Others from approximately the same period reported rates as low as 11% to 25% [20, 21, 26]. These studies consistently reported higher levels of vaccine hesitancy among female and Black respondents, but it decreased with increasing age. Other indicators of low socioeconomic status (e.g., education and income) were also frequently associated with vaccine hesitancy. Serial polls and studies from the Pew Research Center and others show that vaccine hesitancy declined when the FDA issued EUAs for the two COVID-19 vaccines [30–32]. Although these studies provide important national estimates of vaccine hesitancy for the overall population and within subpopulations, online surveys do not capture the variants associated with social context, particularly for rural communities with low and intermittent internet connectivity [33].

In response to the COVID-19 public health emergency, North Carolina Central University (NCCU), a historically black college and university (HBCU), established the Advanced Center for COVID-19 Related Disparities (ACCORD). ACCORD aims to facilitate COVID-19 testing, multidisciplinary research, and messaging directed at historically marginalized populations in several North Carolina counties. A key component of the ACCORD efforts is working in close collaboration with community partners. Unlike online incentivized panels, the ACCORD study investigated vaccine hesitancy in targeted predominately rural and urban communities with entrenched, persistent, socioeconomic and health inequalities. The ACCORD strategy was to enroll a purposive sample at COVID-19 testing events hosted by NCCU. This targeted engagement with communities permitted directly assessing the prevalence and identifying correlates of vaccine hesitancy and whether it changed over time.

## Materials and methods

North Carolina has 100 counties that vary widely with respect to population density, rurality and urbanicity, race/ethnicity, and socioeconomic and health indicators. As an HBCU, NCCU has fostered trusting and collaborative relationships with Black underserved communities for decades. Building on these partnerships, ACCORD facilitated COVID-19 testing and surveying programs in nine counties that represent economically-distressed and Health Research Services Administration (HRSA)-designated medically-underserved areas. ACCORD identified local residents as community facilitators and leveraged existing health resources such as public health departments in each county to garner community support for COVID-19 testing events. ACCORD hosted 32 testing events at locations carefully selected by community facilitators to provide access in otherwise COVID-19 testing deserts. Testing events occurred between August 27, 2020 and December 15, 2020.

ACCORD COVID-19 testing events took place in the parking lots of churches, schools, and similar venues that accommodated drive-through testing. ACCORD partnered with health departments and other service providers to collect nasal swabs for PCR tests. Eligibility criteria to participate in the survey included at least 18 years of age, English or Spanish comprehension, and providing informed consent. NCCU students, faculty and staff greeted individuals in their cars and explained that the university was conducting a survey to better understand the experiences and thoughts about COVID-19 in their community. If they agreed, they received the consent form and survey on a sanitized clipboard with a new ink pen that had never been used (to keep). (Initially, participants were offered sanitized tablets to enter their responses electronically but this was discontinued for a variety of reasons.) All volunteers had their temperatures recorded upon arrival, wore masks at all times, sanitized their hands frequently, and used sanitized clipboards and tablets. Survey participants received a variety of NCCU-branded items (e.g., T-shirts, string bags, and cups) and their names were entered into a monthly raffle for gift cards. The Institutional Review Board at NCCU approved the study.

### Measures

To reduce respondent burden, participants received one of three survey questionnaires that each assessed different topics related to COVID-19 in greater depth. The different surveys assessed psychosocial stress, barriers to COVID-19 testing, contact tracing acceptability, and electronic media use. Each version included a set of core questions. Each survey was clearly marked with a version number (i.e., 1, 2, 3) to ensure that they were evenly distributed. All three versions assessed COVID-19 vaccine hesitancy and one version had additional questions about trusted sources for information about the vaccine.

Vaccine hesitancy was assessed (across all three questionnaire versions) with the question “*Scientists are working on a COVID-19 vaccine*. *Would you get vaccinated against COVID-19 as quickly as possible when the vaccine becomes available*?” Response choices included *yes*, *no*, and *don’t know/not sure*. To explore features of vaccine hesitancy, regardless of participants’ responses, the survey then asked, “*Which of the reasons below would stop you or delay you from getting vaccinated against COVID-19 as soon as the vaccine becomes available*?” Participants could choose multiple responses including: 1) *Don’t believe that vaccines work;* 2) *Have concerns about vaccine safety;* 3) *Do not trust the government about the vaccine;* 4) *Do not trust the medical system; and* 5) *Want others to get the vaccine first*.

The outcome used for regression analysis—vaccine hesitancy—combined the responses *no*, and *don’t know/not sure*. Participants who skipped or declined to answer the question were excluded. This analysis also assessed temporal trends for vaccine hesitancy over the data collection period by segmenting the events into three time periods: August-September, October, and November-December. The month number during which the event occurred was treated as a continuous variable in regression models.

### Analysis

All analyses were conducted using Stata Ver 15 (College Station, TX). The analysis generated descriptive statistics and tabular analysis with Chi Square or Fisher’s exacts tests, estimated the prevalence of vaccine hesitancy, quantified reasons to delay or not get vaccinated, and changes over time. Respondents who skipped or declined to answer question(s) were excluded from most analyses. Logistic regression models were used to identify correlates associated with vaccine hesitancy. We selected variables for the adjusted regression model on the basis of previous studies and whether the number of observations was too small for any single variable to generate meaningful findings. A variable with a small cell size can artificially inflate effect estimates. Also, the multivariable model includes variables for questions that were in each of the three survey versions to maximize sample size.

As a sensitivity analysis, we used general linear models (glm) with a poisson distribution, log link function, and robust standard errors. The effect estimates were somewhat attenuated, but consistent with the logistic regression and do not change interpretation (S1 Table).

## Results

We recruited a purposive sample of 1,004 participants from 34 testing events held at times and places accessible to this otherwise unreachable population. Recruitment of participants varied between 50%-95% of people who sought testing. Most participants (94%) reported their race/ethnicity and remained in the analysis. The majority of participants were Black (59.1%), followed by Whites (26.6%) and Hispanics (14.4%) (Table 1). Females comprised 63.9% of the sample. The median age for Blacks was 57 years and significantly older than Whites (45 years) and Hispanics (37 years).

**Table 1:** Characteristics of ACCORD study respondents by race/ethnicity ACCORD study, August-December 2020.

	White		Black		Hispanic		Total		p-value
	252	(26.6)	560	(59.1)	136	(14.4)	948		
**Age**									
18–29	62	(25.5)	63	(12.0)	25	(20.2)	150	(16.8)	<0.0001
30–39	37	(15.2)	58	(11.0)	53	(42.7)	148	(16.6)	
40–59	74	(30.5)	168	(31.9)	37	(29.8)	279	(31.2)	
≥60	70	(28.8)	238	(45.2)	9	(7.3)	317	(35.5)	
Median (IQR)^a^	45 (29–62)		57 (41–67)		37 (39–46.5)		50 (35–64)		<0.0001
**Gender**									
male	116	(46.2)	186	(33.8)	33	(25.8)	335	(36.1)	<0.0001
female	135	(53.8)	364	(66.2)	95	(74.2)	594	(63.9)	
**Education**									
< high school	9	(3.6)	53	(9.7)	48	(40.)	110	(12.)	<0.0001
high school/GED	53	(21.2)	161	(29.4)	27	(22.5)	241	(26.3)	
some college/trade	77	(30.8)	161	(29.4)	17	(14.2)	255	(27.8)	
bachelor	67	(26.8)	100	(18.3)	19	(15.8)	186	(20.3)	
graduate	44	(17.6)	72	(13.2)	9	(7.5)	125	(13.6)	
**Marital status**									
Married/cohabitate	143	(57.4)	214	(39.3)	73	(60.8)	430	(47.1)	<0.0001
widow	13	(5.2)	63	(11.6)	3	(2.5)	79	(8.6)	
divorce/separated	34	(13.7)	100	(18.3)	16	(13.3)	150	(16.4)	
never married	59	(23.7)	168	(30.8)	28	(23.3)	255	(27.9)	
**Income—annual**									
< $20K	49	(23.2)	142	(30.8)	51	(51.)	242	(31.3)	<0.0001
$20-40K	46	(21.8)	150	(32.5)	30	(30.)	226	(29.3)	
$40-60K	33	(15.6)	91	(19.7)	9	(9.)	133	(17.2)	
>$60K	83	(39.3)	78	(16.9)	10	(10.)	171	(22.2)	
**Residence**									
house	207	(83.5)	369	(68.)	52	(41.9)	628	(68.6)	<0.0001
apartment	18	(7.3)	103	(19.)	25	(20.2)	146	(16.)	
mobile home	23	(9.3)	63	(11.6)	47	(37.9)	133	(14.5)	
other	0		8	(1.5)	0		8	(0.9)	
**Health care**									
Health insurance	204	(83.3)	454	(85.8)	37	(28.9)	695	(77.1)	<0.0001
Trust provider: COVID-19 test information	114	(45.2)	240	(42.9)	44	(32.1)	398	(42.0)	0.030
**Difficulty paying for**									
food	33	(13.1)	85	(15.2)	30	(22.1)	148	(15.6)	0.061
monthly bills	66	(26.2)	194	(34.6)	51	(37.5)	311	(32.8)	0.027
medical care and prescriptions	23	(9.1)	75	(13.4)	26	(19.1)	124	(13.1)	0.020
	**Subset of sample who received questions**								
**Technology**	**153**	** **	**353**	** **	**101**	** **	**607**	** **	
Computer	117	(76.5)	217	(61.5)	35	(34.7)	369	(60.8)	<0.0001
Mobile phone	135	(88.2)	284	(80.5)	70	(69.3)	489	(80.6)	0.001
Internet access	133	(92.4)	261	(80.1)	60	(78.9)	454	(83.2)	<0.0001

^a^ IQR = Interquartile range

Overall, 15.6%, 38.2%, and 13.1% of respondents reported difficulty paying for food, monthly bills and medical care or prescriptions, respectively (Table 1). Whites reported the lowest prevalence of poor socioeconomic markers and Hispanics were markedly poorer than both Blacks and Whites. Half of Hispanics (51%) reported an annual income of <$20K (compared to 23% and 30.8% of Whites and Blacks, respectively), 40% did not complete high school, 37.9% lived in mobile homes, and only 28.9% had health insurance. Hispanics were less likely to trust health care providers for information about COVID-19 testing (32.1%) as compared to 45.2% and 42.9% of Whites and Blacks, respectively.

Disparities with respect to technology and access to the internet were evident among all survey participants. Approximately 39% of respondents did not own a computer. Only 69.3% of Hispanics owned a mobile phone as compared 88.2% of Whites and 80.5% of Blacks. Although internet access in the home was nearly universal among Whites (92.4%), significantly fewer Blacks (80.1%) and Hispanics (78.7%) reported internet access.

### Vaccine hesitancy

Indicators of vaccine hesitancy varied by race/ethnicity (Table 2); only 23.4% of Blacks as compared to 36.1% of Whites and 32.6% Hispanics reported affirmatively that they would get vaccinated as soon as possible. Comparable proportions across all groups (35–36%) were unsure or did not know if they would get the vaccine as soon as possible and 89 respondents (9%) skipped the question. Notably, 10% of Blacks and 18.5% of Hispanics did not answer the question. Excluding participants who skipped the question and combining the responses *no* and *don’t know* classifies 69% of respondents as vaccine hesitant including 62.7%, 74%, and 59.5% of Whites, Blacks, and Hispanics, respectively (Table 2).

**Table 2:** Prevalence and features of vaccine hesitancy stratified by race, ACCORD study, North Carolina August–November 2020.

	**Total sample**								
	**White**		**Black**		**Hispanic**		**Total**		
**Total n by race**	**276**	**(27.7)**	**594**	**(59.6)**	**126**	**(12.7)**	**996**	**(100.)**	
** **	**N**	**col%**	**N**	**col%**	**N**	**col%**			**p-value**
Get COVID-19 vaccine as soon as possible?									
Yes	91	(36.1)	131	(23.4)	44	(32.6)	266	(28.1)	<0.0001
No	64	(25.4)	177	(31.6)	17	(12.6)	258	(27.2)	
don’t know	89	(35.3)	196	(35.)	49	(36.3)	334	(35.3)	
not answered	8	(3.2)	56	(10.)	25	(18.5)	89	(9.4)	
*Vaccine hesitancy* ^a^^,^^b^	*153*	*(62*.*7)*	*373*	*(74*.*0)*	*66*	*(59*.*5)*	*592*	*(68*.*9)*	*<0*.*0001*
Reasons for not or delay getting vaccinated									
doesn’t believe vaccines work	6	(2.4)	47	(8.4)	6	(4.4)	59	(6.2)	
safety concerns	137	(54.4)	296	(52.9)	43	(31.9)	476	(50.3)	<0.0001
mistrust government	45	(17.9)	160	(28.6)	18	(13.3)	223	(23.5)	<0.0001
mistrust medical system	5	(1.9)	38	(14.7)	7	(2.1)	50	(5.8)	
want others to get vaccine first	57	(22.6)	144	(25.7)	23	(17.)	224	(23.7)	
	**Subset of sample who received questions**								
**Total n**	**84**	**(25.4)**	**194**	**(58.6)**	**53**	**(16.)**	**331**	**(100)**	
Trusted sources for vaccine information									
Health websites	27	(36.)	50	(26.7)	8	(15.4)	85	(27.1)	0.01
Healthcare provider	53	(70.7)	126	(67.4)	19	(36.5)	198	(63.1)	<0.0001
Community service organization	21	(28.)	42	(22.5)	10	(19.2)	73	(23.2)	
Pastor or other faith leaders	5	(6.7)	18	(9.6)	6	(11.5)	29	(9.2)	
Received flu shot within 12 months	34	(45.9)	74	(42.)	16	(39.)	124	(42.6)	

^a^ Excludes n = 89 participants who did not answer question.

^b^ Hesitancy combines responses *no* and *don’t know/not sure*.

The most common reason for not- or delaying- getting vaccinated was safety concerns (50.3%). (Table 2). Only 17% of Hispanics wanted others to receive the vaccine first, as compared to 22.6% of Whites and 25.7% of Blacks. Significantly more Blacks (28.6%) mistrusted the government (as compared to 17.9% and 13.3% of Whites and Hispanics, respectively). Among the subset of respondents (n = 311) who received additional questions, trusted sources for information about the vaccine diverged significantly for Hispanics. Only 36.5% of Hispanics trust health care providers for information on COVID-19 as opposed to 70.7% and 67.4% of Whites and Blacks, respectively.

**Fig 1:**
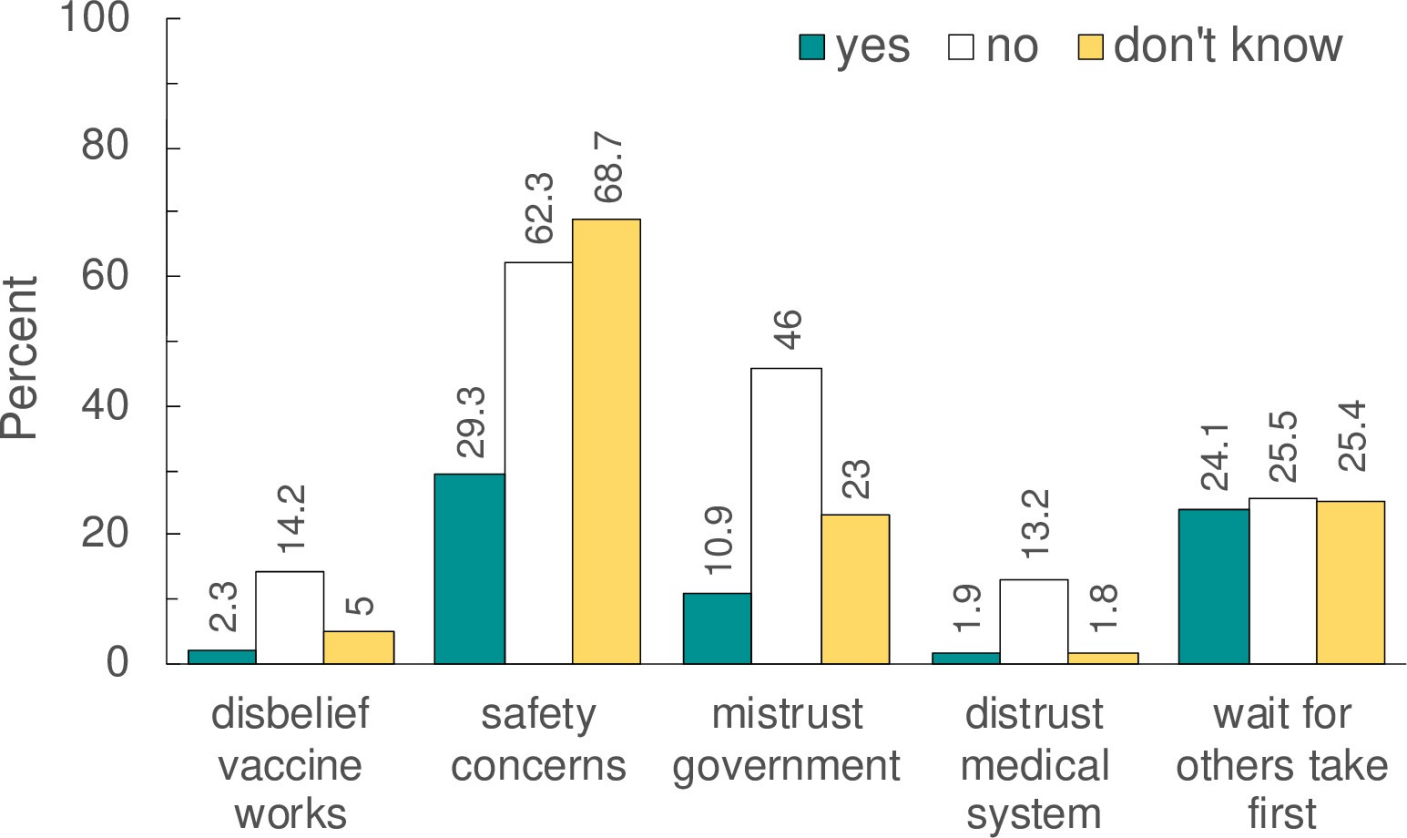
Distribution of reasons to prevent or delay vaccination stratified by participants’ response to getting vaccinated as soon as it became available.

Fig 1 presents the distributions of reasons to prevent or delay vaccination stratified by participants’ responses to getting the vaccine as soon as possible. These results reveal important subtleties. Although 28.1% of respondents reported that they would indeed get the vaccine (Table 2), they nevertheless reported they had safety concerns (30.7%) (Fig 1). Respondents who reported they would *not* get vaccinated were more likely to report that vaccines don’t work (14.7%) and government mistrust (43%). It is important to note that between 24–26% of participants, independent of their vaccine hesitancy (or lack of) wanted others to get the vaccine first (p-value = 0.769).

**Fig 2:**
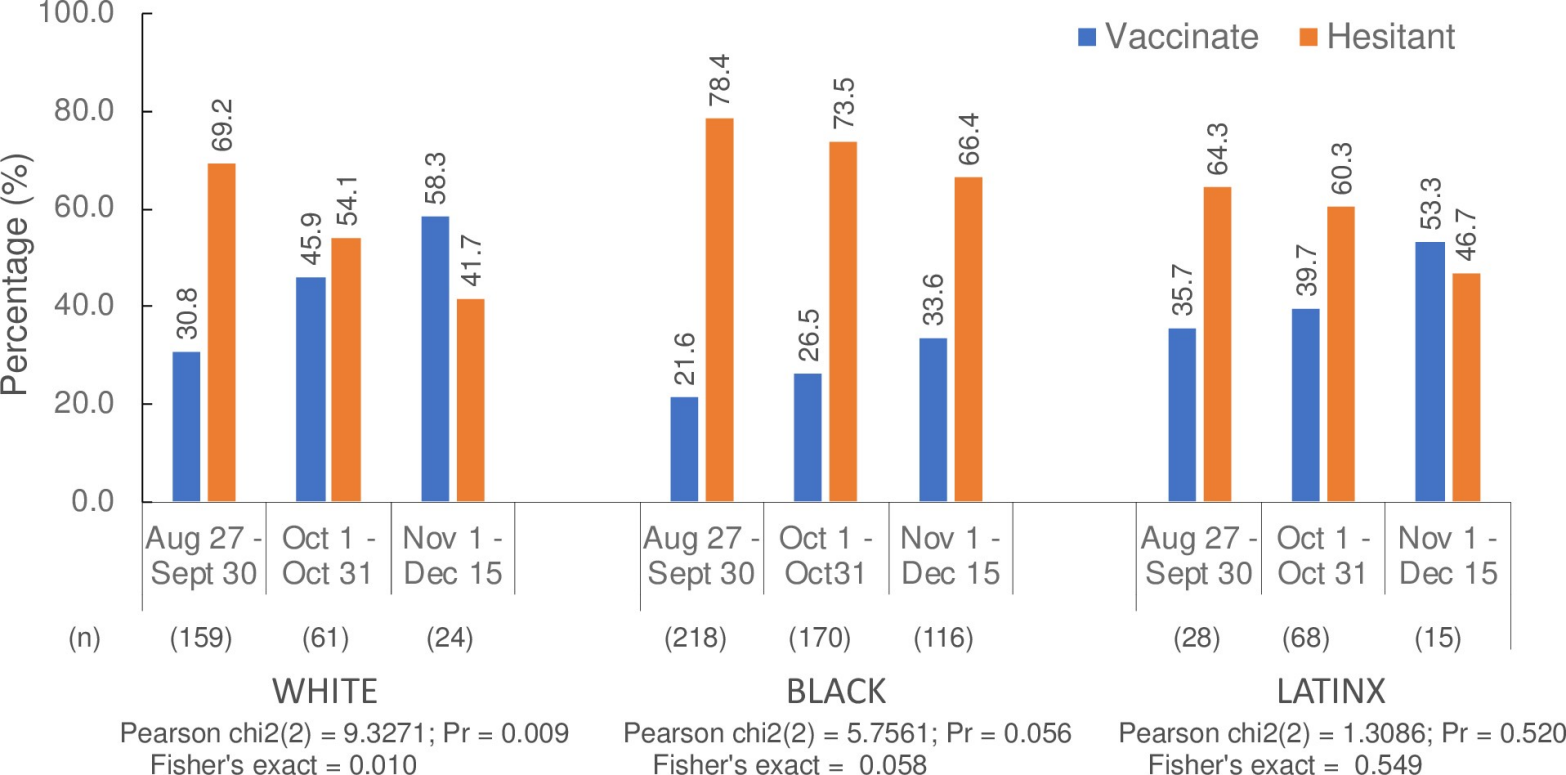
Distribution of vaccine hesitancy over time stratified by race/ethnicity and calendar time.

Fig 2 displays patterns of temporal changes over three intervals of vaccine hesitancy and stratified by race/ethnicity. The prevalence of hesitancy declined over the course of data collection for full sample and each racial/ethnic groups. The prevalence of hesitancy among Whites declined by 27.5 percentage points from a high of 69.2% during September to 41.7% during November-December (p-value = 0.01). While vaccine hesitancy also significantly declined among Blacks, the prevalence was very high during the first interval (78.4%) and declined by only 12 percentage points (66.4%) during November-December. It was not statistically significant for Hispanics because testing events in Hispanic neighborhoods started about one month (October 2020) after data collection started.

### Logistic regression

Table 3 presents the bivariate associations with vaccine hesitancy. Although ownership of mobile phones (OR = 2.12, 95%CI [1.31, 3.43]) and computers (OR = 1.46, 95%CI [1.00, 2.13]), were associated with hesitancy, they were not included in the adjusted model because the questions were asked to 64% of participants (n = 607). While older adults (age ≥65) were less likely to report vaccine hesitancy (OR = 0.70 95%CI [0.50,0.98], Table 3), age was not in the multivariable model because it was highly correlated with race/ethnicity. In the multivariable model (Table 4), Blacks were 1.68(1.16, 2.45) times as likely Whites and females were 1.90 (1.36, 2.64) times as likely as males to report vaccine hesitancy, as well as participants who expressed safety concerns (OR = 4.28 [3.06, 5.97]), government distrust (OR = 3.57 [2.26, 5.63]), or wanting others to get vaccinated first (OR = 1.44, [0.98, 2.11], marginally significant p = 0.062). Hesitancy also persisted to significantly decline over time with each passing month (OR = 0.76 [0.63, 0.92]).

**Table 3:** Bivariate logistic regression—vaccine hesitancy, ACCORD study, NC August-December 2020.

	Vaccinate		Hesistant		Total				
	N	row%	N	row%	N	OR	(95% CI)		p-value
**Race**									
White	91	(37.3)	153	(62.7)	244	ref			
Black	131	(26.0)	373	(74.0)	504	1.69	(1.22	2.35)	0.002
Hispanic	45	(40.5)	66	(59.5)	111	0.87	(0.55	1.38)	0.560
**Gender**									
male	124	(39.9)	187	(60.1)	311	ref			
female	141	(26.2)	398	(73.8)	539	1.87	(1.39	2.52)	<0.0001
**Data collection period**									
Aug-Sept	106	(26.2)	299	(73.8)	405	ref			
October	100	(33.4)	199	(66.6)	299	0.71	(0.51	0.98)	0.036
Nov-Dec	61	(39.4)	94	(60.6)	155	0.55	(0.37	0.81)	0.002
Event month (8–12) (continuous variable)						0.79	(0.67	0.93)	0.004
**Age**									
18–64 years	175	(28.4)	444	(71.5)	615	ref			
≥65 years	72	(36.2)	127	(63.8)	199	0.70	(0.50	0.98	
per 10 year age increase (continuous variable)						0.93	(0.86	1.01)	0.106
**Education**									
< high school	31	(35.6)	56	(64.4)	87	ref			
high school/GED	66	(30.8)	148	(69.2)	214	1.24	(0.73	2.10)	0.420
some college/trade	75	(31.1)	166	(68.9)	241	1.23	(0.73	2.05)	0.441
bachelor	41	(23.4)	134	(76.6)	175	1.81	(1.03	3.17)	0.038
graduate	42	(35.3)	77	(64.7)	119	1.01	(0.57	1.81)	0.960
**Income**									
< 20K	69	(32.7)	142	(67.3)	211	ref			
20-40K	61	(28.6)	152	(71.4)	213	1.21	(0.80	1.83)	0.365
40-60K	28	(21.9)	100	(78.1)	128	1.74	(1.04	2.88)	0.034
>60k	54	(32.9)	110	(67.1)	164	0.99	(0.64	1.53)	0.963
**Marital Status**									
married/cohabitate	129	(33.3)	258	(66.7)	387	ref			
widow	22	(31.)	49	(69.)	71	1.11	(0.65	1.92)	0.699
divorce/separated	40	(29.2)	97	(70.8)	137	1.21	(0.79	1.85)	0.374
never married	65	(27.3)	173	(72.7)	238	1.33	(0.93	1.90)	0.115
**Residence**									
house	175	(30.2)	405	(69.8)	580	ref			
apartment	39	(29.8)	92	(70.2)	131	1.02	(0.67	1.54)	0.928
mobile home	44	(36.7)	76	(63.3)	120	0.75	(0.49	1.13)	0.163
other	2	(28.6)	5	(71.4)	7	1.08	(0.21	5.62)	0.927
**Health insurance**									
Yes	200	(30.8)	450	(69.2)	650	ref			
No	61	(33.5)	121	(66.5)	182	0.88	(0.62	1.25)	0.480
**Difficulty paying for:**								
food	42	(30.7)	95	(69.3)	137	1.02	(0.69	1.52)	0.907
monthly bills	84	(29.5)	201	(70.5)	285	1.12	(0.82	1.53)	0.473
medical care or prescriptions	32	(28.6)	80	(71.4)	112	1.15	(0.74	1.78)	0.538
**Pandemic**									
Know someone who had COVID	106	(28.2)	270	(71.8)	376	1.25	(0.93	1.68)	0.143
**Reasons to prevent or delay vaccine**							
doesn’t believe vaccines work	7	(12.1)	51	(87.9)	58	3.50	(1.57	7.82)	0.002
safety concerns	82	(17.6)	383	(82.4)	465	4.13	(3.03	5.64)	<0.0001
mistrust government	29	(13.7)	183	(86.3)	212	3.67	(2.41	5.61)	<0.0001
want others to get vaccine first	64	(29.1)	156	(70.9)	220	1.13	(0.81	1.59)	0.459
**Technology**	**Subset of sample who received questions (n = 607)**								
internet access	122	(28.0)	313	(72.0)	435	1.28	(0.78	2.10)	0.322
mobile phone	126	(27.0)	341	(73.0)	467	2.12	(1.31	3.43)	0.002
computer	97	(26.8)	265	(73.2)	362	1.46	(1.00	2.13)	0.053

**Table 4:** Multivariable logistic regression–vaccine hesitancy ACCORD study, NC August-December 2020.

	OR	(95% CI)		p-value
**Race**				
White	ref			
Black	1.68	(1.16	2.45)	0.006
Hispanic	1.14	(0.67	1.91)	0.634
**Gender**				
male	ref			
female	1.90	(1.36	2.64)	<0.0001
**Calendar time**				
Event month (8–12)	0.76	(0.63	0.92)	0.004
**Reasons to prevent or delay vaccine**				
safety concerns	4.28	(3.06	5.97)	<0.0001
mistrust government	3.57	(2.26	5.63)	<0.0001
want others to get vaccine first	1.44	(0.98	2.11)	0.062

## Discussion

The ACCORD study enrolled over 1000 participants attending COVID-19 testing events in targeted communities with high racial/ethnic minorities that suffer from health disparities and economic inequalities. The pandemic has intensified historically valid distrust of the government, medical establishment, and probably academic researchers. Our success in conducting this study emanates from the innate trust embedded in the definition of an HBCU, in addition to enlisting local residents as community facilitators who looked like participants, and our on-the-ground approach.

One other study in the US (to date) mirrors our methods [34]. Researchers at the University of California Berkeley have collaborated with Latino farmworker communities for decades. They recruited 1115 farmworkers receiving free COVID-19 tests at clinics and other community events during July–November 2020. Half (52%) of participants reported they would be extremely likely to get vaccinated and 32% were unsure. Most vaccine-hesitant participants reported worry about side effects (~65%). While this study and the ACCORD study reached different marginalized populations, they share similar concerns. Both studies suggest that working within communities where trust is earned is essential for addressing vaccination and the many needs that the COVID-19 pandemic has created or made worse.

Unlike numerous online panel studies [17–28, 35] representative of the US population with internet access and email addresses, we captured vast differences in COVID-19 vaccine hesitancy among historically marginalized populations living in areas that lack technology and reliable internet service [33]. Furthermore, the target population has experienced disproportionally higher rates of COVID-19 infections and deaths. Obtaining nationally representative samples of the US, using rigorous sampling methodology to estimate vaccine hesitancy (or other health conditions) informs national public health policy. We assert such approaches do not translate to the devastation of the pandemic in the communities with the greatest needs.

The estimated prevalence of vaccine hesitancy in national studies ranged between ~11% to 35% whereas in the ACCORD study, it ranged between 42% to 79% depending on the race/ethnicity and data collection interval window. Online surveys tended to skip the questions about reasons not to vaccinate. Such questions were administered to all respondents in our paper survey. It revealed that people who would get the vaccine as soon as possible, nonetheless, had safety concerns and wanted others to get the vaccine first (Fig 2). This finding suggests that seeing others’ reactions to the vaccine is a universal concern for 25% of the population.

Repeated polls report that vaccine hesitancy has dropped over time (yet remains higher among Blacks) [25, 31]. Surveys of health care workers reported more hesitancy (than the public) at the beginning of the pandemic, but as the results from clinical trials were released, hesitancy diminished precipitously [36, 37]. Despite these promising trends, vaccine hesitancy may persist among historically marginalized populations unless substantial resources, funding, messaging, public health activities, and access to vaccination programs are deployed and evaluated.

Intervention research scientists often engage Black pastors and church leaders to promote healthy behaviors [38, 39]. Our findings show that fewer than 10% of respondents sought the advice of the faith leaders for COVID-19 vaccine information. The importance and influence of faith leaders, however, cannot be undermined, ignored, or diminished. Although, the majority of White and Black respondents (62.5%) placed their trust in their health care providers, efforts must be made to collectively utilize community and faith leaders and health providers of color [24] to deliver accurate and reliable information about the vaccine [15]. The findings also demonstrate the need for additional tailored interventions for Hispanics and their health providers to build trust. Misinformation may change attitudes and the appearance of any side effects from the first shot may deter people from receiving their second shot and any booster vaccinations. Providing transparent information about vaccine development, potential side effects, and answering related questions will help ameliorate vaccine hesitancy.

There are limitations of the ACCORD survey. The data originate from a purposive sample recruited from COVID-19 testing events of people who agreed to take the extra time to complete the survey. Although the study design could not determine detailed information about the extent that participants reflect the experiences of others in their communities, the high prevalence of distrust reported is evidence that we reached an otherwise unreachable population.

Logistical constraints prevented collecting COVID-19 test results from participants. That said, *not* collecting testing results likely increased participation and preserved community trust.

## Conclusions

Our target population lived in extremely rural settings and disenfranchised urban neighborhoods. ACCORD’s structure within a large HBCU coupled with endorsement by community trusted facilitators enabled us to reach people where they live, work, and worship. Although vaccine hesitancy continues to plummet in the US as vaccine provision increases, it remains a priority to continue to directly monitor hesitancy in the communities disproportionately affected by COVID-19 –on the ground where needed most.

## Supporting information

S1 Table Multivariable sensitivity analysis. IRR = Incidence Rate Ratio.
(DOCX)

## References

[1] Webb Hooper M, Nápoles AM, Pérez-Stable EJ. COVID-19 and Racial/Ethnic Disparities. *JAMA*. 2020;323(24):2466–2467. doi: 10.1001/jama.2020.8598 32391864 PMC9310097

[2] Clouston SAP, Natale G, Link BG. Socioeconomic inequalities in the spread of coronavirus-19 in the United States: A examination of the emergence of social inequalities. *Soc Sci Med*. 2021;268:113554. doi: 10.1016/j.socscimed.2020.113554 33308911 PMC7703549

[3] Woolf SH, Chapman DA, Lee JH. COVID-19 as the Leading Cause of Death in the United States. *JAMA*. Published online December 17, 2020. doi: 10.1001/jama.2020.24865 33331845 PMC8553021

[4] Woolf SH, Chapman DA, Sabo RT, Weinberger DM, Hill L, Taylor DDH. Excess Deaths From COVID-19 and Other Causes, March-July 2020. *JAMA*. 2020;324(15):1562. doi: 10.1001/jama.2020.19545 33044483 PMC7576405

[5] CDC. Coronavirus Disease 2019 (COVID-19). Centers for Disease Control and Prevention. Published February 11, 2020. Accessed December 23, 2020. https://www.cdc.gov/coronavirus/2019-ncov/covid-data/investigations-discovery/hospitalization-death-by-race-ethnicity.html

[6] Baden LR, El Sahly HM, Essink B, et al. Efficacy and Safety of the mRNA-1273 SARS-CoV-2 Vaccine. *N Engl J Med*. 2020;0(0):null. doi: 10.1056/NEJMoa2035389 33378609 PMC7787219

[7] Polack FP, Thomas SJ, Kitchin N, et al. Safety and Efficacy of the BNT162b2 mRNA Covid-19 Vaccine. *N Engl J Med*. 2020;383(27):2603–2615. doi: 10.1056/NEJMoa2034577 33301246 PMC7745181

[8] Coronavirus (COVID-19) Update: FDA Holds Advisory Committee Meeting to Discuss Authorization of COVID-19 Vaccine Candidate as Part of Agency’s Review of Safety and Effectiveness Data. FDA. Published December 10, 2020. Accessed December 22, 2020. https://www.fda.gov/news-events/press-announcements/coronavirus-covid-19-update-fda-holds-advisory-committee-meeting-discuss-authorization-covid-19

[9] FDA Takes Additional Action in Fight Against COVID-19 By Issuing Emergency Use Authorization for Second COVID-19 Vaccine. FDA. Published December 21, 2020. Accessed December 22, 2020. https://www.fda.gov/news-events/press-announcements/fda-takes-additional-action-fight-against-covid-19-issuing-emergency-use-authorization-second-covid

[10] Committee on Equitable Allocation of Vaccine for the Novel Coronavirus, Board on Health Sciences Policy, Board on Population Health and Public Health Practice, Health and Medicine Division, National Academies of Sciences, Engineering, and Medicine. *Framework for Equitable Allocation of COVID-19 Vaccine*. (Gayle H, Foege W, Brown L, Kahn B, eds.). National Academies Press; 2020:25917. doi: 10.17226/25917 33026758

[11] Dooling K. The Advisory Committee on Immunization Practices’ Updated Interim Recommendation for Allocation of COVID-19 Vaccine—United States, December 2020. *MMWR Morb Mortal Wkly Rep*. 2020;69. doi: 10.15585/mmwr.mm695152e2 33382671 PMC9191902

[12] Weinberg GA, Szilagyi PG. Vaccine Epidemiology: Efficacy, Effectiveness, and the Translational Research Roadmap. *J Infect Dis*. 2010;201(11):1607–1610. doi: 10.1086/652404 20402594

[13] Kwok KO, Lai F, Wei WI, Wong SYS, Tang JWT. Herd immunity–estimating the level required to halt the COVID-19 epidemics in affected countries. *J Infect*. 2020;80(6):e32–e33. doi: 10.1016/j.jinf.2020.03.027 32209383 PMC7151357

[14] Fine P, Eames K, Heymann DL. “Herd Immunity”: A Rough Guide. *Clin Infect Dis*. 2011;52(7):911–916. doi: 10.1093/cid/cir007 21427399

[15] Wood S, Schulman K. Beyond Politics—Promoting Covid-19 Vaccination in the United States. Malina D, ed. *N Engl J Med*. Published online January 6, 2021:NEJMms2033790. doi: 10.1056/NEJMms2033790 33406324

[16] MacDonald NE. Vaccine hesitancy: Definition, scope and determinants. *Vaccine*. 2015;33(34):4161–4164. doi: 10.1016/j.vaccine.2015.04.036 25896383

[17] Pogue K, Jensen JL, Stancil CK, et al. Influences on Attitudes Regarding Potential COVID-19 Vaccination in the United States. *Vaccines*. 2020;8(4):582. doi: 10.3390/vaccines8040582 33022917 PMC7711655

[18] Fisher KA, Bloomstone SJ, Walder J, Crawford S, Fouayzi H, Mazor KM. Attitudes Toward a Potential SARS-CoV-2 Vaccine: A Survey of U.S. Adults. *Ann Intern Med*. 2020;173(12):964–973. doi: 10.7326/M20-3569 32886525 PMC7505019

[19] Kreps S, Prasad S, Brownstein JS, et al. Factors Associated With US Adults’ Likelihood of Accepting COVID-19 Vaccination. *JAMA Netw Open*. 2020;3(10):e2025594. doi: 10.1001/jamanetworkopen.2020.25594 33079199 PMC7576409

[20] Taylor S, Landry CA, Paluszek MM, Groenewoud R, Rachor GS, Asmundson GJG. A Proactive Approach for Managing COVID-19: The Importance of Understanding the Motivational Roots of Vaccination Hesitancy for SARS-CoV2. *Front Psychol*. 2020;11:575950. doi: 10.3389/fpsyg.2020.575950 33192883 PMC7604422

[21] Southwell BG, Kelly BJ, Bann CM, Squiers LB, Ray SE, McCormack LA. Mental Models of Infectious Diseases and Public Understanding of COVID-19 Prevention. *Health Commun*. 2020;35(14):1707–1710. doi: 10.1080/10410236.2020.1837462 33081500

[22] Carpiano RM. Demographic Differences in US Adult Intentions to Receive a Potential Coronavirus Vaccine and Implications for Ongoing Study. *medRxiv*. Published online September 9, 2020:2020.09.07.20190058. doi: 10.1101/2020.09.07.20190058

[23] Reiter PL, Pennell ML, Katz ML. Acceptability of a COVID-19 vaccine among adults in the United States: How many people would get vaccinated? *Vaccine*. 2020;38(42):6500–6507. doi: 10.1016/j.vaccine.2020.08.043 32863069 PMC7440153

[24] Head KJ, Kasting ML, Sturm LA, Hartsock JA, Zimet GD. A National Survey Assessing SARS-CoV-2 Vaccination Intentions: Implications for Future Public Health Communication Efforts. *Sci Commun*. 2020;42(5):698–723. doi: 10.1177/1075547020960463 PMC7520657 38602991

[25] Muñana C, 2020. KFF COVID-19 Vaccine Monitor: December 2020—Methodology. KFF. Published December 15, 2020. Accessed December 27, 2020. https://www.kff.org/report-section/kff-covid-19-vaccine-monitor-december-2020-methodology/

[26] Khubchandani J, Sharma S, Price JH, Wiblishauser MJ, Sharma M, Webb FJ. COVID-19 Vaccination Hesitancy in the United States: A Rapid National Assessment. *J Community Health*. Published online January 3, 2021. doi: 10.1007/s10900-020-00958-x 33389421 PMC7778842

[27] Callaghan T, Moghtaderi A, Lueck JA, et al. Correlates and disparities of intention to vaccinate against COVID-19. *Soc Sci Med*. Published online January 4, 2021:113638. doi: 10.1016/j.socscimed.2020.113638 33414032 PMC7834845

[28] Bokemper SE, Huber GA, Gerber AS, James EK, Omer SB. Timing of COVID-19 vaccine approval and endorsement by public figures. *Vaccine*. 2021;39(5):825–829. doi: 10.1016/j.vaccine.2020.12.048 33390295 PMC7744009

[29] Szilagyi PG, Thomas K, Shah MD, et al. National Trends in the US Public’s Likelihood of Getting a COVID-19 Vaccine—April 1 to December 8, 2020. *JAMA*. 2021;325(4):396. doi: 10.1001/jama.2020.26419 33372943 PMC7772743

[30] Daly M, Robinson E. *Willingness to Vaccinate against COVID-19 in the US*: *Longitudinal Evidence from a Nationally Representative Sample of Adults from April–October 2020*. Public and Global Health; 2020. doi: 10.1101/2020.11.27.20239970 33773862 PMC7883746

[31] Intent to Get a COVID-19 Vaccine Rises to 60% as Confidence in Research and Development Process Increases. Pew Research Center; 2020. Accessed December 22, 2020. https://www.pewresearch.org/science/2020/12/03/intent-to-get-a-covid-19-vaccine-rises-to-60-as-confidence-in-research-and-development-process-increases/

[32] Elon University Poll. Elon University. Accessed January 22, 2021. https://www.elon.edu/u/elon-poll/

[33] Digital gap between rural and nonrural America persists Pew Research Center. Accessed December 22, 2020. https://www.pewresearch.org/fact-tank/2019/05/31/digital-gap-between-rural-and-nonrural-america-persists/

[34] Mora AM, Lewnard JA, Kogut K, et al. Impact of the COVID-19 Pandemic and Vaccine Hesitancy among Farmworkers from Monterey County, California. *medRxiv*. Published online January 1, 2020:2020.12.18.20248518. doi: 10.1101/2020.12.18.20248518

[35] Ruiz JB, Bell RA. Predictors of intention to vaccinate against COVID-19: Results of a nationwide survey. *Vaccine*. 2021;39(7):1080–1086. doi: 10.1016/j.vaccine.2021.01.010 33461833 PMC7794597

[36] Meyer MN, Gjorgjieva T, Rosica D. Healthcare worker intentions to receive a COVID-19 vaccine and reasons for hesitancy: A survey of 16,158 health system employees on the eve of vaccine distribution. *medRxiv*. Published online January 1, 2020:2020.12.19.20248555. doi: 10.1101/2020.12.19.20248555

[37] Shekhar R, Sheikh AB, Upadhyay S, et al. COVID-19 Vaccine Acceptance among Health Care Workers in the United States. *Vaccines*. 2021;9(2):119. doi: 10.3390/vaccines9020119 33546165 PMC7913135

[38] Corbie-Smith G, Goldmon M, Roman Isler M, et al. Partnerships in Health Disparities Research and the Roles of Pastors of Black Churches: Potential Conflict, Synergy, and Expectations. *J Natl Med Assoc*. 2010;102(9):823–831. doi: 10.1016/s0027-9684(15)30680-5 20922927 PMC3056456

[39] Williams RM, Glanz K, Kegler MC, Davis E. A Study of Rural Church Health Promotion Environments: Leaders’ and Members’ Perspectives. *J Relig Health*. 2012;51(1):148–160. doi: 10.1007/s10943-009-9306-2 19960262

