# Supplementary material for: COVID-19 vaccine hesitancy in underserved communities of North Carolina
Source: PLoS One. 2021 Nov 1;16(11):e0248542. doi: 10.1371/journal.pone.0248542 (PMC8559933; doi:10.1371/journal.pone.0248542)
Supplement: S1 Table — IRR = Incidence Rate Ratio. (DOCX) [file pone.0248542.s001.docx]

**S1 Table Multivariable sensitivity analysis**

|  | **IRR*** | **(95% CI)** | | **p-value** |
| --- | --- | --- | --- | --- |
| **Race** |  |  |  |  |
| White | ref |  |  |  |
| Black | 1.14 | (1.03 | 1.26) | 0.012 |
| Hispanic | 1.03 | (0.86 | 1.22) | 0.777 |
|  |  |  |  |  |
| **Gender** |  |  |  |  |
| Male | ref |  |  |  |
| Female | 1.19 | (1.08 | 1.31) | 0.000 |
|  |  |  |  |  |
| **Calendar time** |  |  |  |  |
| Event month | 0.93 | (0.88 | 0.98) | 0.008 |
|  |  |  |  |  |
| **Reasons to prevent or delay vaccine** | |  |  |  |
| safety concerns | 1.50 | (1.36 | 1.66) | 0.000 |
| mistrust government | 1.28 | (1.17 | 1.38) | 0.000 |
| want others to get vaccine first | 1.05 | (0.96 | 1.15) | 0.288 |
|  |  |  |  |  |

*Incidence rate ratio
